# Karyotype reinvestigation does not confirm the presence of two cryptic species and interspecific hybridization in the *Polyommatus* (*Agrodiaetus*) *damocles* complex in the Crimea (Lepidoptera, Lycaenidae)

**DOI:** 10.3897/CompCytogen.v13i3.46777

**Published:** 2019-10-17

**Authors:** Vladimir A. Lukhtanov, Konstantin A. Efetov, Alexander V. Dantchenko

**Affiliations:** 1 Department of Karyosystematics, Zoological Institute of the Russian Academy of Sciences, Universitetskaya nab. 1, St. Petersburg 199034, Russia Zoological Institute, Russian Academy of Sciences St. Petersburg Russia; 2 Department of Entomology, St. Petersburg State University, Universitetskaya nab 7/9, St. Petersburg 199034, Russia St. Petersburg State University St. Petersburg Russia; 3 Department of Biological Chemistry and Laboratory of Biotechnology, V. I. Vernadsky Crimean Federal University, Lenin blvd. 5/7, Simferopol 295051, Russia V. I. Vernadsky Crimean Federal University Simferopol Russia; 4 Faculty of Chemistry, Lomonosov Moscow State University, GSP-1, Leninskiye Gory 1/11, Moscow119991, Russia Zoological Institute, Russian Academy of Sciences Saint Petersburg Russia

**Keywords:** Biodiversity, chromosome, hybrids, meiosis, karyosystematics, taxonomy

## Abstract

The karyotype of the blue butterflies from the Angarskiy Pass (Crimea), previously attributed to Polyommatus (Agrodiaetus) poseidon (Herrich-Schäffer, 1851), was re-examined. In all 19 studied individuals, we found the haploid chromosome number n = 26, including 7 pairs of relatively large and 19 pairs of relatively small chromosomes. According to the chromosome number and karyotype structure, the studied population does not differ from P. (A.) damocles krymaeus (Sheljuzhko, 1928) from the eastern part of the Crimean Mountains. This result does not confirm the previously formulated hypotheses, according to which (1) two morphologically similar but karyologically different species, P. (A.) poseidon and P. (A.) damocles krymaeus, occur sympatrically in the Crimea and (2) there is hybridization between these taxa on the Angarskiy Pass. Thus, only three species of the subgenus Agrodiaetus Hübner, 1822 have been reliably established for the Crimea: P. (A.) damone pljushtchi Lukhtanov & Budashkin, 1993, P. (A.) damocles krymaeus (Sheljuzhko, 1928) and P. (A.) ripartii budashkini Kolev & de Prins, 1995.

## Introduction

The subgenus Agrodiaetus Hübner, 1822 of the genus *Polyommatus* Latreille, 1804 is a diverse and taxonomically difficult group of blue butterflies of the subtribe Polyommatina (Talavera et al. 2013), consisting of a large number of species weakly differentiated in morphology (Eckweiler and Bozano 2016). At the same time, the subgenus demonstrates a high level of karyotypic differentiation with respect to the chromosome number (Przybyłowicz et al. 2014, Lukhtanov 2015, Vershinina and Lukhtanov 2017), chromosome size (Lukhtanov and Dantchenko 2002) and number of chromosomes bearing ribosomal DNA clusters (Vershinina et al. 2015). Therefore, cytogenetic studies are an absolutely necessary (although not always sufficient) approach for solving many problems of the taxonomy in the subgenus Agrodiaetus (de Lesse 1960, Lukhtanov and Dantchenko 2017, Lukhtanov and Shapoval 2017, Vishnevskaya et al. 2018).

Karyotypes of the Crimean *Agrodiaetus* were studied by Kandul (1997) who indicated three species for this territory: P. (A.) damone pljushtchi Lukhtanov & Budashkin, 1993 (with the haploid chromosome number, n = 67), P. (A.) ripartii budashkini Kolev & de Prins, 1995 (n = 90) and P. (A.) poseidon (Herrich-Schäffer, 1851). The latter species, according to Kandul, is represented by two karyomorphs in the Crimea. One of them, determined by Kandul as P. (A.) poseidon krymaeus (Sheljuzhko, 1928) [currently known as P. (A.) damocles krymaeus (Sheljuzhko, 1928), see Eckweiler and Bozano 2016], has n = 26. It is quite widespread in the southern Crimea from the Angarskiy Pass in the west to the village of Kurortnoye in the east. Another karyomorph, determined by Kandul (1997) as P. (A.) poseidon poseidon (Herrich-Schäffer, [1851]), has n = 19. According to Kandul, the latter karyomorph was found in four individuals collected by K. A. Efetov on the Angarskiy Pass in 1992 (see the Discussion section for the alternative possible origin of these specimens). In addition, abnormal meiotic metaphase plates were found in the specimens from the Angarskiy Pass presumably indicating hybridization between P. (A.) damocles krymaeus and P. (A.) poseidon (Kandul 1997).

Since these chromosomal morphs (n = 26 and n = 19) were reported to inhabit in sympatry, it could be assumed that they belong to different species. This is a plausible assumption given that (1) the distribution areas of P. (A.) damocles and P. (A.) poseidon overlap in Turkey (Eckweiler and Bozano 2016), (2) P. (A.) poseidon is distributed more widely and represented on its northern edge by extremely local populations which remain unknown until now even on the well-studied territories (Lukhtanov and Tikhonov 2015), and (3) the larval foodplant of P. (A.) poseidon also belongs to the genus *Hedysarum* Linnaeus, 1753 (Fabaceae) as it was reported before for P. (A.) damocles krymaeus (Budashkin 1990, Dantchenko 1995, 1997, Dantchenko, unpubl.). The presence of hybrid individuals does not preclude this assumption, since their meiosis was reported to be abnormal (Kandul 1997).

To test the hypotheses about the two karyomorphs and interspecific hybridization, we re-examined the karyotypes of the blues from the same population (Crimea, the Angarskiy Pass) that was previously studied by Kandul (1997).

## Material and methods

Adult males were collected by K. A. Efetov on the Angarskiy Pass of the Crimean Mountains exactly in the same place where in 1993 (erroneously cited by Kandul as "1992") the Kandul’s material (Kandul 1997) was collected. The collection of the new material was carried out during two summer seasons: in 1997 and in 1998.

Testes were extracted from the butterfly abdomens and fixed in a mixture of glacial acetic acid and 96% ethyl alcohol (1 : 3). The fixed material was stored at + 4°C for 5–6 months. The testes were stained with 2% orcein acetic acid for 8–30 days as previously described (Lukhtanov 2017, Efetov et al. 2004, 2015 and references therein). The stained material was placed in a drop of 40% lactic acid on a glass slide. The testes were macerated with thin pins. The slide was covered with a coverslip and the macerated testes were squashed between the two glasses. Excess lactic acid was removed with filter paper. Karyotypes were studied in 19 individuals. For determination of karyotype parameters, 175 metaphase plates of the highest quality and 2 cells at the stage of diakinesis were selected. Cells in which the chromosomes were not located on the same plane, as well as cells with overlapping or touching chromosomes and/or bivalents, were rejected and not used for analysis. Haploid chromosome number (n) was counted at metaphase I (MI), metaphase II (MII) and diakinetic cells. In some cases, diploid chromosome number (2n) was counted in spermatogonial mitotic metaphase plates and atypical meioses (see: Lorković 1990, Lukhtanov and Dantchenko 2017 for an explanation and illustration of atypical meiosis in Lepidoptera).

## Results and discussion

Butterflies of the P. (A.) damocles species complex have a single generation in the Crimea (Budashkin 1990), and therefore the adults can be encountered there from the first to the last days of July. In 1997, only the karyomorph n = 26 was found among the butterflies collected at the end of July (i.e. at the end of the flying period). Since it was impossible to exclude that the karyomorph n = 19 has a different phenology, and butterflies with this karyotype fly earlier, then in 1998, individuals were collected in all parts of the flying period: from the first to the last days of July. However, the karyomorph n = 19 was never detected.

**Figures 1–4.**
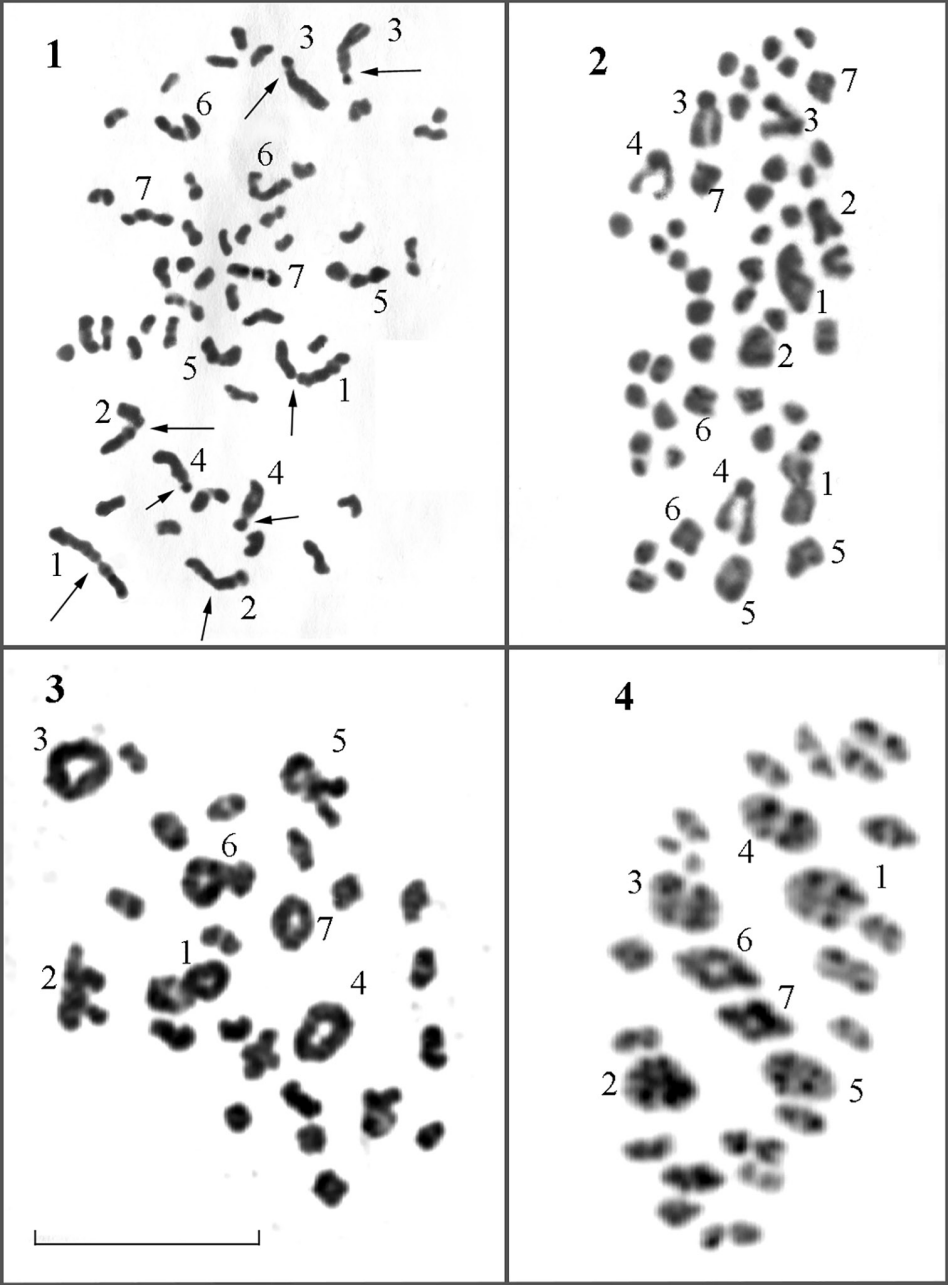
Karyotype in male mitosis and meiosis of Polyommatus (Agrodiaetus) damocles krymaeus from the Angarskiy Pass (Crimea). Numbers from 1 to 7 show the largest chromosome pairs in mitosis and the largest bivalents in meiosis. **1** metaphase of spermatogonial mitosis, 2n = 52 **2** early anaphase of spermatogonial mitosis, 2n = 52 **3** diakinesis, n = 26 **4** MI, n = 26. Scale bar: 10 μm.

Summing up the analysis of the samples collected on the Angarskiy Pass, 26 bivalents were found in all studied individuals in all cells at the stages of MI and diakinesis, and 26 chromosomes at the stage of MII. The karyotype at these stages is sharply asymmetric and includes 7 relatively large and 19 relatively small bivalents. Diploid chromosome number 2n = 52 was found in spermatogonial mitosis and atypical meiosis. No variation in the chromosome number was observed. Therefore, we do not confirm the numbers n = 25 and n = 27 reported for this population by Kandul (1997) along with the modal number n = 26. The information obtained is summarized in the Table 1. Photos of karyotypes are shown in the Figures 1–4.

**Table 1.** Chromosome numbers found in P. (A.) damocles krymaeus (Crimea, the Angarskiy Pass).

Code of specimen	Dates of collection	Chromosome number	Quantity and type of studied cells
1997-A	25.07.1997	n = 26	27 MI
1997-B	26.07.1997	n = 26	1 MI
1997-C	26.07.1997	n = 26	2 MI
1997-D	31.07.1997	n = 26	1 MI
1998-1	2.07.1998	n = 26	4 MI
1998-2	2.07.1998	n = 26	1 MI
1998-4	12.07.1998	n = 26	7 MI
1998-4	12.07.1998	n = 26	2 MII
1998-4	12.07.1998	2n = 52	2 atypical divisions
1998-4	12.07.1998	2n = 52	2 mitotic metaphases
1998-5	14.07.1998	n = 26	13 MI
1998-6	14.07.1998	n = 26	20 MI
1998-6	14.07.1998	n = 26	20 MII
1998-7	15.07.1998	n = 26	2 MI
1998-8	15.07.1998	n = 26	9 MI
1998-9	15.07.1998	n = 26	16 MI
1998-11	15.07.1998	n = 26	8 MI
1998-11	15.07.1998	n = 26	2 diakinetic cells
1998-12	15.07.1998	n = 26	7 MI
1998-13	15.07.1998	n = 26	4 MI
1998-14	21.07.1998	n = 26	4 MI
1998-51	23.07.1998	n = 26	5 MI
1998-54	23.07.1998	n = 26	7 MI
1998-55	23.07.1998	n = 26	9 MI
1998-55	23.07.1998	n = 26	4 MII

**Figures 5–8.**
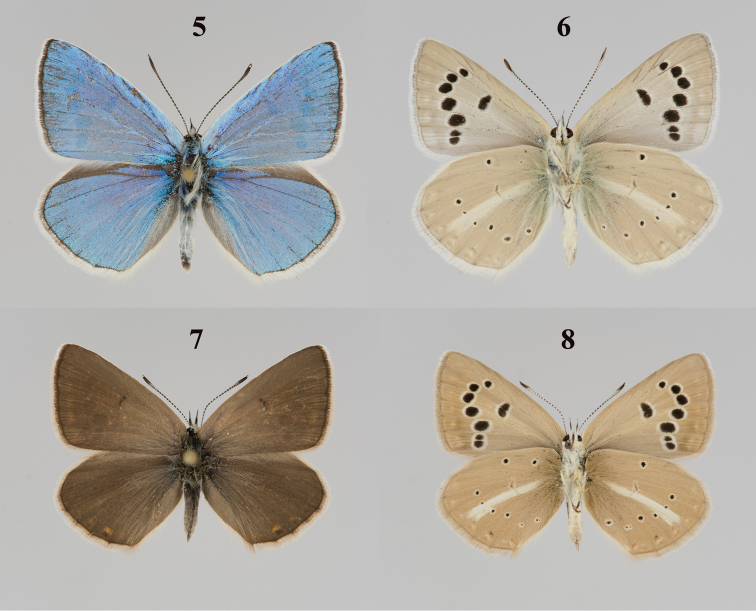
Polyommatus (Agrodiaetus) damocles krymaeus, Crimea, Karadagh, Legener Mt. **5** male, upperside **6** male, underside **7** female, upperside **8** female, underside.

Analyzing the information at our disposal, as well as butterflies collected by K. A. Efetov on the Angarskiy Pass in 1993 (Kandul’s material, cited by him as “1992”) and 1997–1998 (our material), we conclude that, most likely, there are no blue butterflies of the subgenus Agrodiaetus with the karyotype n = 19 in the Crimea. Our conclusion is based on the following:

1) the presence of the karyomorph n = 19 is not confirmed by several studies, all 19 individuals studied from the Angarskiy Pass have n = 26;

2) males of P. (A.) poseidon and P. (A.) damocles are not identical in their appearance. Males of P. (A.) damocles have a darker blue coloration, and their veins on the wing upperside possess black scales (Eckweiler and Bozano 2016). If these two species are present on the Angarskiy pass, we expect to find both phenotypes here. However, this is not the case. The individuals E92003, E92012, E92014 and E92015, for which the karyotype n = 19 was reported (Kandul 1997), are identical in their external morphology to all other individuals from the Angarskiy Pass, for which n = 26 was established. All these individuals have the phenotype of P. (A.) damocles krymaeus (Figs 5–8).

Additionally, our data do not confirm the conclusion (Kandul 1997) about chromosome number variation in P. (A.) damocles krymaeus from n = 25 to n = 27. We found the number n = 26 to be stable;

3) the conclusion about hybridization between the two putative chromosomal races was based on the analysis of the metaphase plates of insufficient quality (see: Kandul 1997: fig. 6). In the given micrographs, it is impossible to distinguish between bivalents and multivalents; we therefore conclude that the assumption of the presence of multivalents on these metaphase plates remains unsupported by the data.

In the end, we assume that the karyomorph n = 19 from the Angarskiy Pass does not actually exist and is an artifact, although it is rather difficult to explain the possible origin of this error. It cannot be excluded that it resulted from an accidental mix-up between the chromosomal preparations (but not between the butterfly samples) of P. (A.) poseidon from Turkey (n = 19) and P. (A.) damocles krymaeus from the Crimea (n = 26), since they were processed in parallel (see Kandul and Lukhtanov 1997).

In terms of the chromosome number and karyotype structure, the studied population found on the Angarskiy Pass is the same as that from the eastern Crimea (Sudak region) (Figs 5–8), although the wings of males of the Angarskiy Pass population are somewhat darker. We assign both these populations to the taxon originally described as “*Lycaena damone* Ev. *krymaea* (subsp. nov.) Sheljuzhko, 1928” (type locality: Agarmysh mountain near Stary Krym town, see Dantchenko 1997). In turn, this taxon is a subspecies of P. (A.) damocles to which it is most similar in terms of morphology (Dantchenko 1997), karyotype structure (Lukhtanov et al. 1997) and molecular characters (Kandul et al. 2007).
